# Autologous Fibroblast Cells in Platelet‐Rich Plasma Injection as a Novel Treatment for Inactive En Coup de Sabre Deformity

**DOI:** 10.1002/ccr3.71272

**Published:** 2025-10-17

**Authors:** Sona Zare, Alireza Jafarzadeh, Maryam Nouri, Elaheh Lotfi, Solmaz Zare, Nastaran Kabiri Samani, Mohammad Ali Nilforoushzadeh

**Affiliations:** ^1^ Skin and Stem Cell Research Center Tehran University of Medical Sciences Tehran Iran; ^2^ Pars Fundamental Bio Structure Company Tehran Iran; ^3^ Stem Cell and Regenerative Medicine Institute, Sharif University of Technology Tehran Iran; ^4^ Department of Mechanical Engineering Sharif University of Technology Tehran Iran; ^5^ Skin Repair Research Center, Jordan Dermatology and Hair Transplantation Center Tehran Iran; ^6^ Department of Dermatology, Hazrat Fatemeh Hospital, School of Medicine, Iran University of Medical Sciences Tehran Iran; ^7^ Laser Application in Medical Sciences Research Center Shahid Beheshti University of Medical Sciences Tehran Iran

**Keywords:** autoimmune skin disease, en coup de sabre, fibroblast cells, linear morphea, platelet‐rich plasma, skin regeneration

## Abstract

Morphea is a chronic autoimmune condition characterized by sclerosis and scar‐like changes in the skin and underlying tissues. En coup de sabre represents a rare and severe linear variant of morphea, primarily affecting the frontoparietal scalp and forehead, with a higher prevalence among children and women. The disease often leads to significant cosmetic and functional impairments, posing therapeutic challenges due to its unpredictable course and varying responses to conventional treatments. Current management strategies include topical steroids, calcineurin inhibitors, systemic therapies, such as methotrexate, and ultraviolet (UV) therapy. Additionally, interventions like fat grafting and hyaluronic acid injections have demonstrated some efficacy in restoring tissue volume and improving skin texture. This case report explores an innovative approach using autologous fibroblast cell injection combined with platelet‐rich plasma (PRP) as a novel therapeutic option for a 40‐year‐old woman diagnosed with inactive‐phase en coup de sabre. After harvesting fibroblast cells from a superficial skin biopsy and isolating PRP through centrifugation, the combined solution was administered via three monthly subcutaneous injections. No adverse effects were observed throughout the treatment period. At a 3‐month follow‐up, significant improvements were noted in skin elasticity, hydration, and overall cosmetic appearance. Ultrasound imaging revealed enhanced dermal thickness and density, while cutometric and colorimetric assessments confirmed increased skin viscoelastic properties and brightness. The promising results observed in this case suggest that the combination of autologous fibroblasts and PRP may offer a safe, effective, and minimally invasive therapeutic alternative for managing en coup de sabre morphea. However, larger studies and controlled clinical trials are essential to validate these findings, optimize treatment protocols, and further understand the underlying mechanisms driving tissue regeneration and repair in morphea.

**Summary**

- This case report highlights the promising results of combining autologous fibroblast cells with platelet‐rich plasma (PRP) injections as a novel, minimally invasive treatment for inactive‐phase en coup de sabre morphea, showing improvements in skin elasticity, thickness, and overall appearance, warranting further investigation in larger studies.

## 1 Introduction

Morphea, or localized scleroderma, is a rare autoimmune condition primarily affecting the skin and soft tissue, predominantly seen in women [1]. It follows a relapsing–remitting course characterized by alternating phases of inflammation—manifesting as pain, pruritus, erythema, and edema—and fibrosis, marked by dense collagen deposition and yellow‐to‐white plaques. If left untreated, morphea can lead to permanent atrophy and lasting deformity [2].

The pathophysiology of morphea is multifactorial, with immune and fibrotic abnormalities playing central roles. Genetic predisposition, vascular aberrations, trauma, and other environmental triggers are also considered contributing factors [3, 4].

Conventional treatments for active, isolated lesions typically include topical steroids, calcineurin inhibitors, and UV therapy. For more severe or refractory cases, systemic therapies such as methotrexate or mycophenolate mofetil are commonly employed [5].

In recent years, alternative therapies have gained attention. Dermal fat grafting and hyaluronic acid injections have shown effectiveness in improving scalp deformities and addressing inactive scars [6, 7, 8]. Additionally, PRP has emerged as a potential therapeutic option, with reported positive outcomes likely attributed to its high concentration of growth factors that promote tissue remodeling and regeneration [9, 10].

Autologous fibroblast injections have also been explored in dermatology for various applications due to their regenerative properties. In this article, we present a case of a female patient with en coup de sabre morphea successfully treated with a novel combination therapy involving autologous PRP and fibroblast injections.

## 2 Case History/Examination

A 40‐year‐old woman presented to our dermatology clinic with a two‐year history of a progressive linear depression on her forehead. While she did not report any pain, itching, or discomfort, her primary concern was the cosmetic appearance of the affected area, which she felt had worsened over time.

**FIGURE 1:**
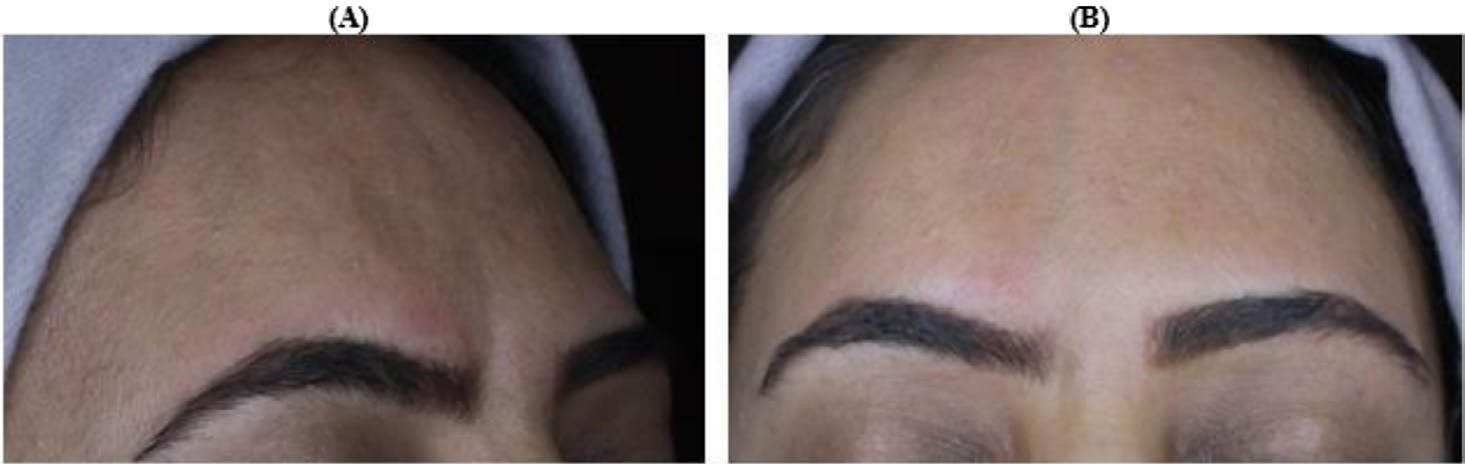
Baseline clinical presentation of en coup de sabre morphea. (A) Right‐sided view showing a distinct linear depression along the patient's forehead, characteristic of en coup de sabre morphea. (B) Frontal view highlighting the extent and location of the atrophic lesion prior to treatment.

On physical examination, a well‐defined linear vertical skin depression, approximately 7 cm in length, was observed on the right medial side of her forehead, consistent with an en coup de sabre pattern (Figure 1). The lesion exhibited skin atrophy and mild hypopigmentation, with no signs of erythema, induration, or active inflammation. There was no evidence of associated neurological or ophthalmic abnormalities upon thorough evaluation.

**FIGURE 2:**
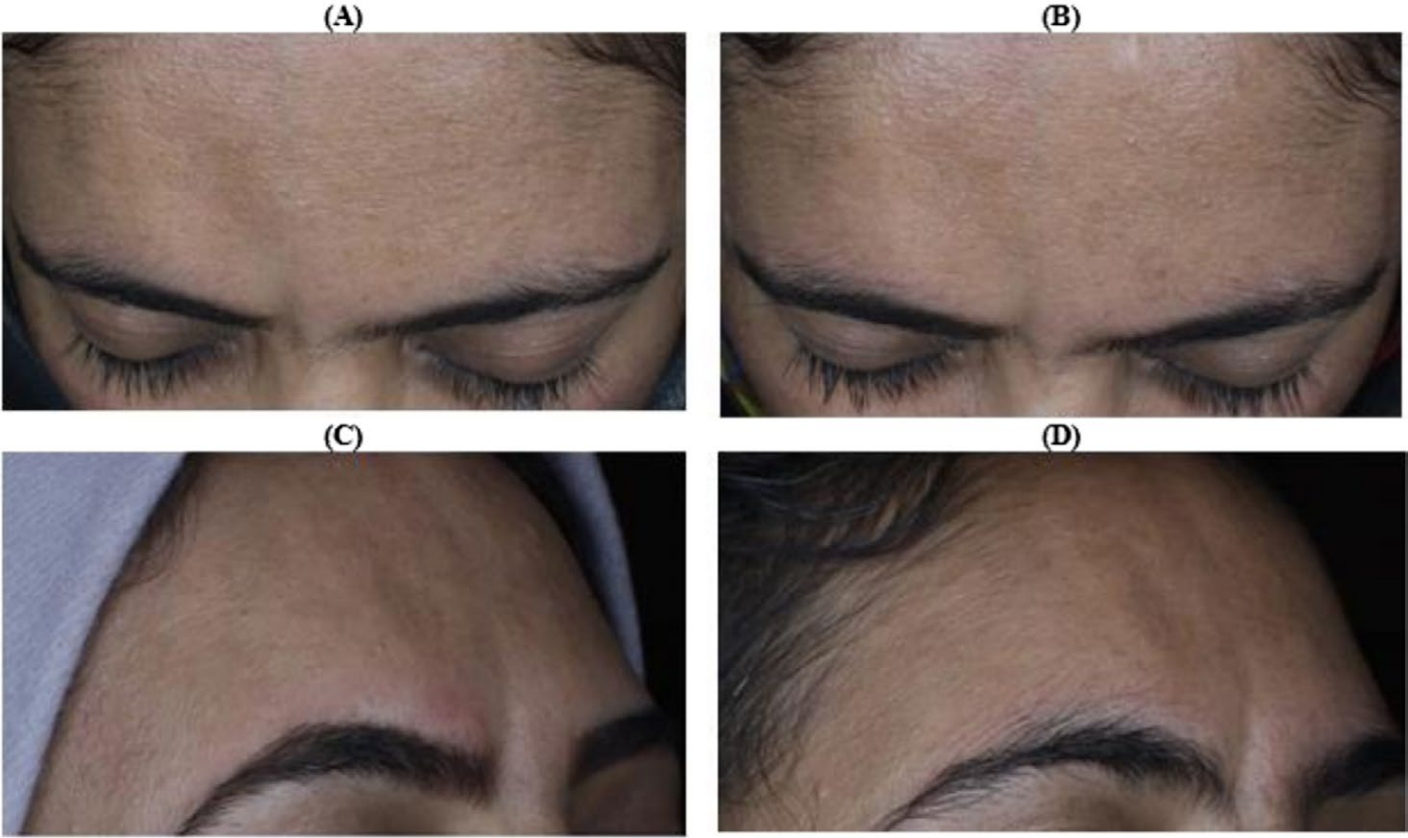
Comparison of baseline and 3‐month post‐treatment clinical images. (A, B) Straight‐on frontal views at baseline and 3 months post‐treatment demonstrating visible improvement in skin volume, texture, and contour. (C, D) Right‐side profile views showing notable filling of the linear depression and enhanced skin smoothness following autologous fibroblast‐PRP therapy.

The patient had no history of trauma, autoimmune disorders, or previous medical or cosmetic treatments for her condition. Additionally, no family history of similar dermatological conditions was reported.

## 3 Methods

A biopsy was taken from the lesion, and histopathological analysis confirmed the diagnosis of en coup de sabre, revealing marked dermal fibrosis, epidermal atrophy, and absence of inflammatory cell infiltration.

To address the patient's cosmetic concerns, a novel autologous fibroblast–PRP therapy was initiated. Following informed consent, a punch biopsy was performed to harvest a small section of superficial dermis from the retroauricular region (posterior to the patient's ear), a site chosen for its minimal sun exposure and higher fibroblast viability. Prior to biopsy, 2% lidocaine was administered intradermally for local anesthesia.

The epidermis was first separated from the dermis by incubation in Dispase II solution (2.4 U/mL; Sigma‐Aldrich, USA) at 4°C overnight. Subsequently, the dermal tissue was enzymatically digested using Collagenase Type I (1 mg/mL; Sigma‐Aldrich, USA) in Dulbecco's Modified Eagle Medium (DMEM; Gibco, USA) at 37°C for 3–4 h to dissociate the dermal fibroblasts. After digestion, the solution was filtered through a 70‐μm cell strainer to obtain a single‐cell suspension. The cells were centrifuged at 1500 rpm for 5 min, resuspended in DMEM supplemented with 10% fetal bovine serum (FBS; Gibco, USA) and 1% penicillin–streptomycin, and cultured in a humidified incubator at 37°C with 5% CO₂.

Fibroblasts at passage 2 were harvested and suspended in freshly prepared platelet‐rich plasma (PRP) solution prior to injection.

Simultaneously, PRP was prepared using a commercially available kit from Pars Fundamental Bio Structure Company, Tehran, Iran. The patient's whole blood was collected and transferred into sterile PRP preparation tubes. The tubes were centrifuged at 1500 Revolutions Per Minute (RPM) for 8 min, and the plasma layer was carefully transferred to a plain tube. A second centrifugation was then performed at 3800 rpm for 8 min. Following centrifugation, the upper two‐thirds of the plasma was discarded, and the remaining plasma was mixed with the platelet pellet at the bottom to create the PRP concentrate.

The cultured autologous fibroblasts were suspended in the freshly prepared PRP under sterile conditions. A total of 5 mL of the fibroblast‐PRP solution was injected subcutaneously into the lesion using a grid‐like injection pattern to ensure even distribution. This procedure was repeated at 1‐month intervals for a total of three sessions to enhance cellular response and tissue remodeling.

## 4 Conclusion and Results

**FIGURE 3:**
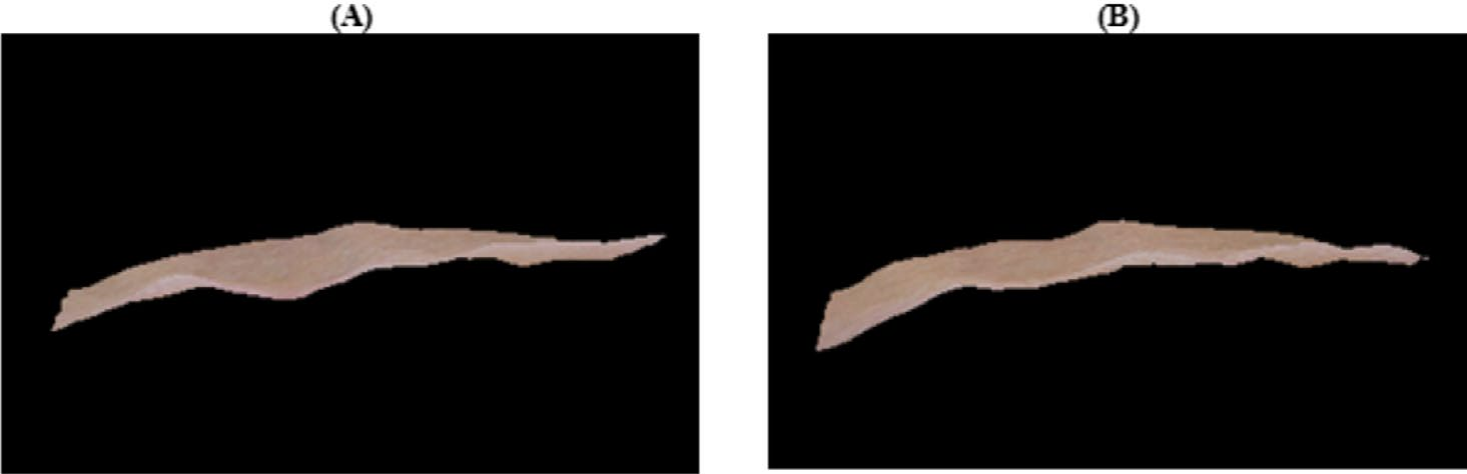
Three‐dimensional imaging of the affected area. Baseline (A) and 3‐month follow‐up (B) 3D reconstructions illustrating volumetric improvements, softening of the lesion margins, and enhanced skin surface uniformity after treatment.

Epidermal hydration, as assessed through corneometry, did not show significant improvement following the treatment. Similarly, Mexametry results, which measure skin pigmentation and erythema, indicated no considerable differences between the pre‐ and post‐treatment evaluations. These findings suggest that while the intervention may not have directly impacted surface hydration or pigmentation, it did have notable effects on the deeper structural and mechanical properties of the skin (see Table 1, Figures 1, 2, 3).

**TABLE 1:** Intervention outcomes at baseline and 3‐months follow up.

Outcome	Pre‐treatment	3‐months follow up
Corneometer	48	50
Colorimeter	11	16
Mexameter	N/A	N/A
Melanin	296.37	290.67
Erythema	419.33	422.00
Cutometer	N/A	N/A
R2	0.7821	0.9759
R5	0.4482	0.6936
R7	0.3486	0.4863

*Note:* Corneometer: Measures skin hydration levels. Colorimeter: Measures skin brightness. Mexameter: Measures melanin (pigmentation) and erythema (redness) levels. Cutometer: Measures skin elasticity parameters: R2 (Overall elasticity); R5 (Net elasticity); R7 (Skin resilience).

**FIGURE 4:**
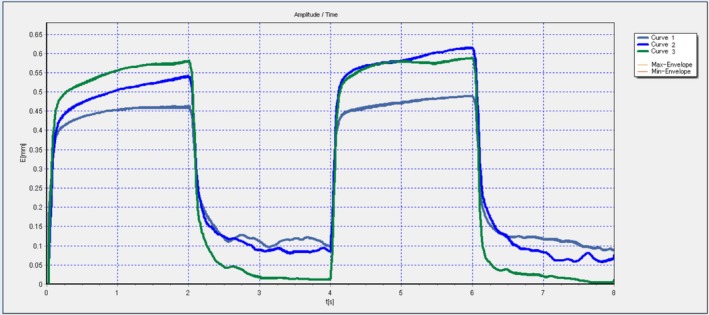
Cutometry assessment of skin elasticity. Graphical representation of skin elasticity parameters (R2, R5, R7) pre‐treatment and post‐treatment. Substantial increases in all parameters reflect improved skin firmness, elasticity, and mechanical resilience after therapy.

In contrast, cutometry assessments, which measure the viscoelastic properties of the skin, demonstrated substantial improvements across all three key parameters: R2 (overall elasticity), R5 (net elasticity), and R7 (skin resilience). These enhancements indicate improved skin firmness, elasticity, and the ability to return to its original state after deformation, reflecting a positive impact on the dermal extracellular matrix and collagen architecture (see Figure 4).

Skin brightness, evaluated using colorimetry, demonstrated a measurable increase after the injections. This improvement suggests enhanced skin quality and light reflectivity, likely resulting from structural changes in the dermis and improved tissue hydration at a deeper level.

**FIGURE 5:**
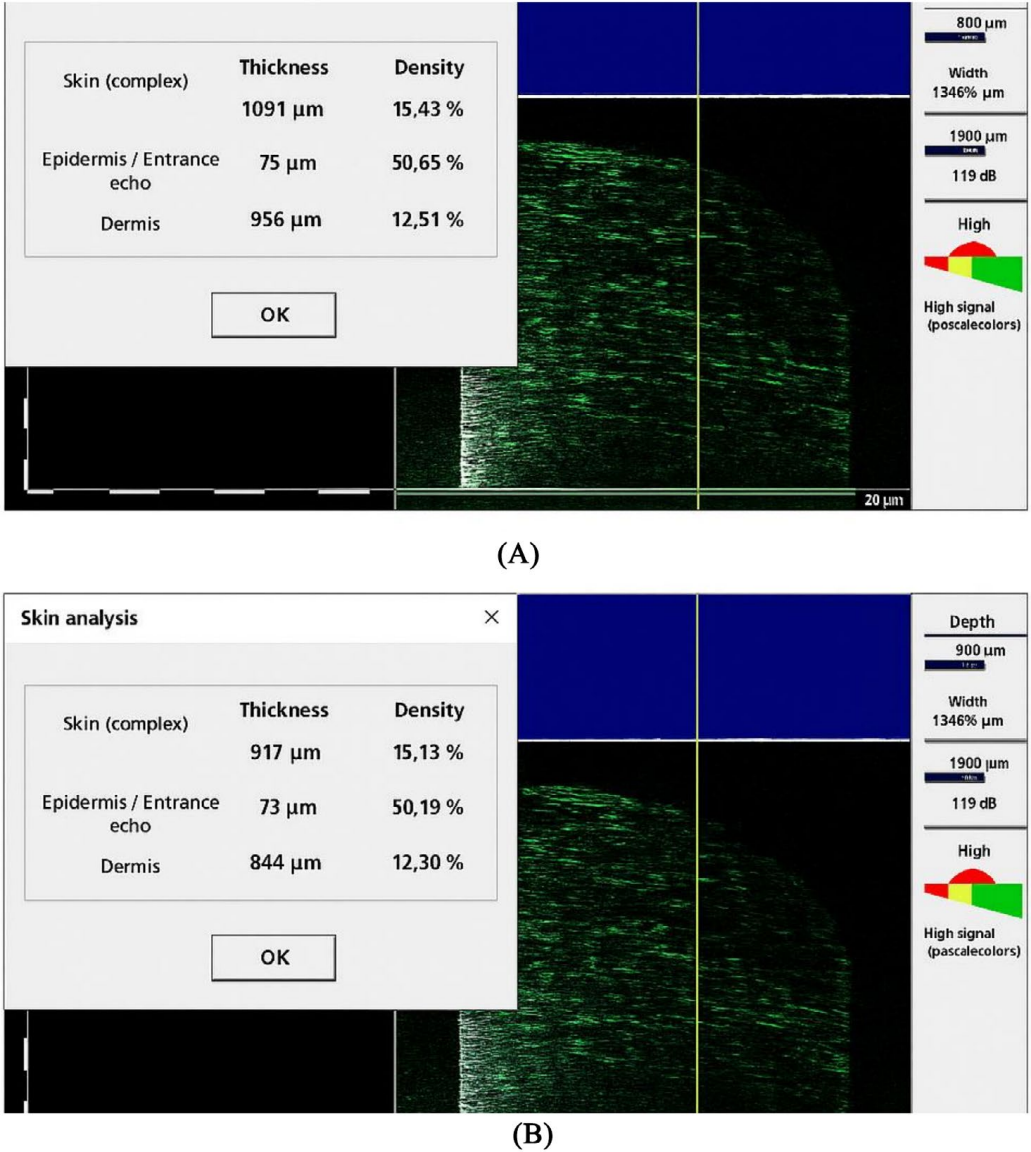
Sonographic evaluation of skin thickness and density. (A) Baseline ultrasound showing reduced dermal and epidermal thickness and lower density. (B) Post‐treatment ultrasound at 3 months demonstrating increased thickness and echogenicity, indicating tissue remodeling and regeneration.

Furthermore, ultrasound imaging was employed to objectively evaluate the thickness and density of the epidermal and dermal layers of the treated skin. The results indicated considerable enhancements in both parameters, with increased skin thickness and improved dermal density noted in comparison to baseline measurements (Table 2, Figure 5). These findings are indicative of effective tissue remodeling and the deposition of new extracellular matrix components facilitated by fibroblasts and PRP growth factors.

**TABLE 2:** Sonographic skin thickness and density measurements of the affected area at baseline and 3‐month follow‐up.

Outcome	Pre‐treatment	3‐month follow‐up
*Thickness (μm)*		
Epidermis layer	65	75
Dermis layer	844	956
Complete thickness	909	1031
*Density (%)*		
Epidermis layer	39.8	50.98
Dermis layer	9.69	11.63
Complete thickness	11.91	14.54

*Note:* Sonographic measurements were performed on the affected area (en coup de sabre lesion) at baseline and at 3 months post‐treatment. No measurements from the contralateral (unaffected) side were included. Thickness: Measured in micrometers (μm) for epidermis, dermis, and total skin. Density: Echogenicity percentage indicating structural integrity of the epidermal and dermal layers.

Collectively, these objective measurements—cutometry, colorimetry, and ultrasound imaging—corroborate the visible clinical improvements observed in the patient, providing quantitative evidence for the efficacy of the treatment.

## 5 Discussion

Untreated morphea can result in irreversible structural and cosmetic damage, making effective intervention crucial to mitigate its long‐term consequences. In this case report, we described a novel therapeutic approach combining intralesional PRP and autologous fibroblast injections in a patient with inactive‐phase en coup de sabre morphea. The outcomes demonstrated promising improvements in both skin texture and elasticity, suggesting this treatment modality as a potential therapeutic option.

While the precise mechanism underlying the observed improvements remains unclear, existing evidence highlights the role of autoimmunity and fibroblast dysregulation in the pathogenesis of morphea. Studies have shown that lymphocytic infiltration and elevated cytokine levels play a critical role in disease progression [11]. Elevated Th2 lymphocyte activity leads to increased secretion of interleukin‐4 (IL‐4) and transforming growth factor‐beta (TGF‐β), both of which stimulate fibroblast activation and excessive collagen deposition [12]. Additional cytokines, including IL‐13, IL‐12, and interferon‐gamma, further contribute to fibrotic changes [13].

Another proposed mechanism suggests that vascular endothelial damage in the early stages of morphea triggers cytokine release, which in turn recruits lymphocytes. These lymphocytes release additional cytokines, perpetuating the fibrotic response [14].

PRP, which is rich in growth factors and bioactive proteins, has been widely studied for its ability to enhance tissue repair and regeneration [15]. On the other hand, fibroblasts, derived from mesenchymal cells, are essential for maintaining the extracellular matrix (ECM) and facilitating wound healing [16]. Their regenerative potential has been successfully applied across various dermatological conditions, including scar healing, alopecia treatment, and skin repair in amputee stump sites [17].

Furthermore, fibroblasts have demonstrated immunomodulatory properties, including the ability to inhibit T‐cell activity and aggregation. In an experimental study by Jalili et al., fibroblast injections in alopecia areata mouse models, an autoimmune T‐cell‐mediated disorder, resulted in reduced CD4+ and CD8+ lymphocyte infiltration and lower levels of pro‐inflammatory cytokines [18].

The combined use of autologous fibroblasts and PRP in our study was based on the complementary mechanisms of action of these two therapies. PRP, rich in growth factors such as platelet‐derived growth factor (PDGF), transforming growth factor‐beta (TGF‐β), and vascular endothelial growth factor (VEGF), primarily stimulates tissue remodeling, angiogenesis, and collagen synthesis. On the other hand, autologous fibroblasts contribute directly by producing extracellular matrix components and exhibiting immunomodulatory effects that can counteract the fibrotic process characteristic of morphea [10].

While PRP alone has been previously used to promote tissue regeneration in various dermatological disorders, its effects are often transient due to the short‐lived nature of growth factor activity. Similarly, fibroblast therapy alone has demonstrated promise in improving scar quality and dermal architecture but may require multiple sessions and extended time frames to achieve substantial clinical improvements [17].

By combining these two therapies, we aimed to harness the immediate regenerative stimulus provided by PRP along with the sustained, structural remodeling capabilities of fibroblasts, thus offering a synergistic effect [10]. This combined approach potentially leads to more durable and enhanced tissue restoration compared to either treatment alone.

Compared to previous studies, [10, 17] reported moderate improvements in skin texture with PRP monotherapy in sclerotic skin conditions, while Shams et al. [17] demonstrated gradual but significant improvements in scar remodeling using autologous fibroblast injections. However, neither study reported rapid or marked improvements in dermal thickness and elasticity within a short follow‐up period as observed in our case. Furthermore, to our knowledge, no previous studies have investigated the use of combined autologous fibroblasts and PRP therapy specifically for morphea, making our report a novel contribution to the field.

These findings suggest that the combination therapy could offer a superior therapeutic strategy for morphea by simultaneously targeting vascular, fibrotic, and regenerative pathways.

The observed improvement in our patient may be attributed to the combined action of PRP‐derived growth factors and the immunomodulatory effects of fibroblasts. This combination likely disrupted the pathological fibrotic pathways, enhanced cellular repair mechanisms, and improved the structural integrity of the affected skin.

To the best of our knowledge, this is the first documented case exploring the combined use of autologous fibroblasts and PRP for inactive‐phase morphea. While the results are encouraging, larger‐scale studies with controlled clinical trials are necessary to validate these findings, optimize treatment protocols, and better understand the underlying mechanisms driving tissue regeneration in morphea.

## 6 Limitations

One major limitation of our study is the relatively short follow‐up period of 3 months. Given the slow and insidious progression of en coup de sabre morphea, a longer follow‐up duration would be necessary to fully assess the stability and durability of the treatment outcomes. Future studies with extended observation periods are warranted to validate these preliminary findings.

## Author Contributions

**Sona Zare:** conceptualization, methodology, project administration, supervision. **Alireza Jafarzadeh:** writing – original draft, writing – review and editing. **Maryam Nouri:** software, supervision. **Elaheh Lotfi:** conceptualization, investigation, methodology. **Solmaz Zare:** visualization, writing – original draft. **Mohammad Ali Nilforoushzadeh:** conceptualization, project administration, supervision. **Nastaran Kabiri Samani:** writing – review and editing.

## Disclosure

Transparency Declaration: Authors declare that the manuscript is honest, accurate, and transparent. No important aspect of the study is omitted.

## Ethics Statement

The researchers were committed to and adhered to the principles of the Helsinki Convention and the Ethics Committee of the Iran University of Medical Sciences at all stages.

## Consent

After providing the necessary explanations, written informed consent was obtained from the patient regarding the submission of their clinical condition to medical journals. Additionally, the patient has been assured that their name and personal details will be kept confidential by the authors.

## Conflicts of Interest

The authors declare no conflicts of interest.

## Data Availability

The data that support the findings of this study are available from the corresponding author upon reasonable request.
